# Putting your money where your mouth is: Geographic targeting of World Bank projects to the bottom 40 percent

**DOI:** 10.1371/journal.pone.0218671

**Published:** 2019-06-21

**Authors:** Hannes Öhler, Mario Negre, Lodewijk Smets, Renzo Massari, Željko Bogetić

**Affiliations:** 1 German Development Institute, Bonn, North Rhine-Westphalia, Germany; 2 World Bank, Washington, D.C., United States of America; 3 LICOS Centre for Institutions and Economic Performance, KU Leuven, Flemish Brabant, Belgium; 4 Independent consultant, Washington, D.C., United States of America; 5 Independent Evaluation Group, World Bank, Washington, D.C., United States of America; Universitat de Valencia, SPAIN

## Abstract

The adoption of the shared prosperity goal by the World Bank in 2013 and Sustainable Development Goal 10, on inequality, by the United Nations in 2015 should strengthen the focus of development interventions and cooperation on the income growth of the bottom 40 percent of the income distribution. This paper contributes to the incipient literature on within-country allocations of development institutions and assesses the geographic targeting of World Bank projects to the bottom 40 percent. Bivariate correlations between the allocation of project funding approved over 2005–14 and the geographical distribution of the bottom 40 as measured by survey income or consumption data are complemented by regressions with population and other potential factors affecting the within-country allocations as controls. The correlation analysis shows that, of the 58 countries in the sample, 41 exhibit a positive correlation between the shares of the bottom 40 and World Bank funding, and, in almost half of these, the correlation is above 0.5. Slightly more than a quarter of the countries, mostly in Sub-Saharan Africa, exhibit a negative correlation. The regression analysis shows that, once one controls for population, the correlation between the bottom 40 and World Bank funding switches sign and becomes significant and negative on average. This is entirely driven by Sub-Saharan Africa and not observed in the other regions. Hence, the significant and positive correlation in the estimations without controlling for population suggests that World Bank project funding is concentrated in administrative areas in which more people live (including the bottom 40) rather than in poorer administrative areas. Furthermore, capital cities receive disproportionally high shares of World Bank funding on average.

## Introduction

The World Bank established overarching twin goals in 2013: ending poverty by 2030 and sharing prosperity. The second goal is new and its basic metric is growth in the real incomes of the bottom 40 percent of the income distribution of the population (the bottom 40) in each country. While the first is a global goal, the second is country-specific. These twin goals are part of a wider international development agenda and are intimately related to United Nations Sustainable Development Goals (SDGs) 1 and 10, respectively, which have been adopted by the global community [1]. In particular, aiming at an income growth of the bottom 40 at a rate higher than the national average coincides with target 1 of Sustainable Development Goal 10. It is defined as the shared prosperity premium and can help reduce inequality over time [2–3].

The adoption of the shared prosperity goal should strengthen the targeting of World Bank interventions to the bottom 40 within countries. Importantly in this context, the increased availability of disaggregated spatial data in recent years has made analyses of the geographic targeting of project funding possible. Such analyses can help to improve efficiency and maximize the poverty- and inequality-reducing effects of development programs [4–6]. Nevertheless, the few studies that exist so far on the within-country targeting of aid are mostly limited to one particular country at a time [7–9]. Briggs [10,11] and Öhler and Nunnenkamp [12] are exceptions. The latter use different development indicators—infant mortality, maternal health, malnutrition—and find no need-sensitive World Bank aid allocation within 27 countries. Briggs [10] finds that World Bank and African Development Bank aid within 17 countries in Africa flows to areas where a greater proportion of the richer population lives (as measured by the possession of assets and the quality of housing) [10]. Briggs [11], on the other hand, uses various measures for poverty down to individual grid cells (50 kilometer × 50 kilometer), most importantly night-time light [11]. He finds that, in up to 52 African countries, better-off areas receive more aid from the African Development Bank and the World Bank relative to poorer areas. However, research on poverty measurement shows that night-time light can be considered an imprecise proxy for poverty at best [13,14]. For instance, Engstrom, Hersh, and Newhouse [13] find that night-time light only explains 10 percent of the variation in poverty headcounts in Sri Lanka.

This study adds value to this growing literature in three important ways. First, we use income and consumption survey data across countries to identify the subnational distribution of the bottom 40. Such data are typically considered as “ground truth” data, i.e., information provided by direct observation, in poverty measurement studies (e.g. [14]). Indeed, official poverty estimates by the World Bank are based on these survey data [3]. Using survey data—instead of proxies—to gauge the targeting will arguably yield more precise estimates. Second, our coverage of recipient countries is larger (58 countries) and not limited to African countries. This allows us to perform a targeting analysis at the regional level. Third, we offer a comprehensive discussion of conceptual issues and operational factors that need to be taken into account when analyzing the geographic targeting of development aid. We consider some of these factors in the regression analysis, i.e., remoteness, conflict, the distribution of domestic public expenditure and the presence of other donors. Accounting for these factors may reveal a different picture with respect to the poverty orientation of development aid. In particular, efficiency and risk considerations may provide valid reasons for donors not to allocate aid to where the poor live. Furthermore, the concentration of government expenditure or aid of other donors in some (poor) areas may prompt the World Bank to focus on other (less poor) areas. Finally, we differentiate between different types of projects (education and health projects versus transportation, energy and mining projects) in the regression analysis.

This paper investigates whether investment projects of the World Bank flow to administrative areas where the bottom 40 are located. To achieve this, we merge a geospatial dataset on the subnational allocation of World Bank investment projects to the geographic distribution of the bottom 40. We then analyze the relationship between World Bank funding and the bottom 40 using both correlations and regression analysis with specific controls.

While this sheds some light on how the World Bank allocates its projects with respect to where the bottom 40 live, the analysis does not provide a measure of deviation from an ideal allocation pattern. Indeed, there is no general ideal aid allocation pattern. Different types of aid aim at different outcomes and there exist a wide arrange of valid criteria donors may choose to drive their allocations, e.g., return to investments, reduction of the poverty headcount or reduction of inequality. Generally speaking, aid can aim at raising the consumption of (poor) households or be used to fund investments. Clements and Kramer [15] discuss whether the World Bank should focus on boosting consumption or funding investments. Carter [16] argues that donors should concentrate on the former in growth-stagnant recipient countries if their objective is to maximize welfare. While we can expect donors to allocate projects intended to raise consumption to areas where the low-income populations live, aid-funded investments, on the other hand, may not require locations near to poor populations in order to have an impact on them. Clear cases of this are the production and provision of energy or any project with positive general equilibrium effects on the poor. In these cases, a negative correlation between the allocation of project funding and the distribution of the bottom 40 does not necessarily indicate poor targeting, but it rather creates a burden of proof on donors to explain their allocation decisions.

The correlation analysis indicates that, of the 58 countries in the sample, 41 show a positive correlation between the shares of the bottom 40 and World Bank funding, and almost half of these show a correlation above 0.5. Of the total sample, slightly more than a quarter of the countries, mostly in Sub-Saharan Africa, exhibit a negative correlation. The regression analysis gives these promising results a more nuanced character. It shows that, once we control for population, the correlation between the bottom 40 and World Bank funding switches sign and becomes significant and negative on average. This is entirely driven by Sub-Saharan Africa and not observed in the other regions. Hence, the significant and positive correlation in the estimations without controlling for population is indicative that World Bank project funding is concentrated in administrative areas in which more people live (including the bottom 40) rather than in poorer administrative areas. Furthermore, capital cities receive disproportionally high shares of World Bank funding on average.

The rest of the paper is organized as follows: Section 2 discusses conceptual issues and operational factors that have to be taken into account when analyzing the geographic targeting of development aid within countries. Section 3 introduces data and methodology, while section 4 presents the correlation and regression results. Section 5 concludes.

## Background and context

An analysis of the geographic targeting of aid within countries requires the consideration of a number of conceptual issues and operational factors. On the one hand, efficiency and risk considerations, general equilibrium effects and donor coordination may provide valid reasons for donors not to allocate aid to where the poor live. On the other hand, political economy considerations may be at play preventing donors from implementing an optimal allocation from a technocratic point of view. Other issues inherent to development aid (i.e., fungibility, aid modalities), information constraints and the choice of the target population further complicate matters. We discuss each of these factors below.

Alongside the targeting of low-income populations, efficiency considerations may play a role in a donor’s decision on the allocation of development assistance within countries. Donors may shy away from difficult environments with weak local institutions and entrenched forms of poverty where the expected returns to aid are low [8]. Physical access also likely influences the within-country geographical allocation of aid. Two opposing forces are at play in this case. First, more accessible areas will tend to perform better economically and typically present higher levels of income. Second, the costs of delivering aid to more remote areas are higher, and therefore aid may become less efficient. Furthermore, security considerations are likely to affect the allocation of aid in countries where security risks are an issue. This presents a clear trade-off as areas exhibiting less security tend also to be poorer so that allocation patterns may positively correlate with more prosperous, safer areas.

Donors are also likely to take into account general equilibrium effects when deciding on the within-country allocation of projects intended to fund investments. For example, energy and infrastructure projects may be located in areas with a low share of the poor, but may have high general equilibrium impacts on the poor [17]. By contrast, the World Bank [18] argues that certain services, such as those related to health care, education, and sanitation should be provided to people in need independently of their location in a country because of the goal of universal coverage in these sectors.

Furthermore, coordination and division of labor among donors within recipient countries are likely to influence the pattern of geographical allocations across individual donors participating in the coordination effort [19]. Indeed, it is plausible that the World Bank has stepped in or out of subnational administrative areas on the basis of agreements with other key donors. However, there might also be important synergies in the joint geographical presence of donor organizations in recipient countries. For example, clustering can have important practical benefits in logistics, security, enhanced local capacities, more leverage on local authorities, and even a greater social awareness of aid practices across officials, communities, and beneficiaries.

Apart from these arguably justified considerations, political economy factors may prevent what would be an optimal aid allocation from a technocratic point of view. Kirk [20] provides an example on the side of the recipient government and highlights that the allocation of World Bank aid within India “has been strongly conditioned by states’ political clout with the central government, owing to their ruling parties’ ties to the central coalition.” However, Nunnenkamp, Öhler, and Sosa Andrés’s [8] empirical analysis does not support this claim. Also Dreher et al. [21] do not find evidence for favoritism in the case of World Bank funding in assessing the influence of the birth areas of country leaders on the amount of funding these areas receive. On the donors’ side, the lower visibility of remote areas versus bigger cities (specially the capital) may affect the allocation of projects and result in a concentration of resources in the latter. However, efficiency and general equilibrium considerations as discussed above may also constitute good reasons for such an allocation pattern.

There are also a couple of issues inherent to development aid that further complicate matters in geographic targeting analyses. First, aid has been found to be at least partly fungible across sectors within countries [22–24]. Insofar as aid is fungible, it may not be possible to target specific groups or affect the income distribution because governments tend to adjust their own spending according to the aid investments they receive. Likewise, the use by a donor of information on government budgetary allocations across administrative areas can also affect the aid patterns of the donor. Thus, a government may adapt its allocations in response to the aid it receives from the international donor community, or, the other way around, donors may allocate aid to those areas where government investments relative to needs are lowest. For this reason, determining the direction of causality in allocations is difficult.

Related to this, the allocation decisions of donors are supposed to be driven by the preferences and development challenges of the recipient countries. The World Bank relies on a country partnership framework to operationalize this approach [25]. The framework is designed to help identify the key objectives and development results through which the World Bank intends to support a country in its efforts to reduce poverty and boost shared prosperity. In preparing a country partnership framework, the World Bank starts from the recipient country's own vision, but aims to select a program that is aligned with the twin goals.

Finally, not all aid modalities are able to target a particular population within a country. In particular, budget support is typically provided to central governments and therefore tends to benefit broader government reform programs and institution building. As such, it cannot be easily tied to specific geographic areas within a country.

This paper spotlights the bottom 40, who are the focus of the World Bank’s second corporate goal. It thus follows the specific, albeit somewhat arbitrary choice made by the World Bank and the international community (in target 1 of SDG 10) that growth in the incomes of the bottom 40 is particularly relevant for the overall economic growth and welfare of societies. One may apply different criteria to assess the targeting of the bottom 40. One might consider either the absolute number of the bottom 40 or the share of the bottom 40 in the population of an administrative area. On the one hand, relatively populous areas with below-average shares of the bottom 40 may receive above-average resources to exert a greater impact on the large absolute number of the bottom 40 in these areas. On the other hand, areas with higher shares of the bottom 40 in their populations may be particularly supported by donors aiming at reducing geographic inequality within countries. On top of this, one may argue that the bottom 20 or bottom 10 percent of the income distribution are more relevant in middle-income countries (where the extreme poor only constitute a small share of the population) if donors are primarily focused on reducing poverty rather than income inequality.

Apart from different targeting criteria, a couple of measurement issues arise when analyzing the geographic targeting of aid projects. First, geographic targeting is arguably most critical in countries where the geographic income distribution is highly uneven. In this case, the gains from targeting are highest (see, e.g., [5]). At the other extreme, in the case of a uniform income distribution across areas, aid projects need not be targeted to specific areas but a simple population-based allocation appears sufficient. Second, some projects may be designed in a way to benefit exclusively or primarily the low-income population in an area. The location of such a project in a relatively rich area may not be seen as problematic or poverty-insensitive. Finally, imperfect local information on the geographic distribution of lower-income populations may prevent proper targeting. While many recipient governments and donors have a general idea where the low-income population is located, local income estimates may differ from expectations. Given the infrequency of surveys in low-income countries and the fact that small area poverty mapping is a relatively new technology and requires substantial technical skill, the necessary information for adequate targeting is limited in many developing countries.

## Data and methodology

For the purpose of the examination of geographic targeting, we use data on the bottom 40 based on representative household surveys in 58 countries on which sufficiently disaggregated geospatial data are available. More specifically, this study has relied on the Global Monitoring Database, a harmonized survey collection produced by the World Bank’s Poverty and Equity Global Practice (internal database). These surveys contain a welfare indicator (income or consumption), a geographic identifier, and a sample weight for each household. Income and consumption aggregates are built following Deaton and Zaidi [26], Ferreira et al. [27] and World Bank [28]. The variables allow us to calculate the number of individuals belonging to the bottom 40 in each first-level administrative division.

Subnational information on the locations of World Bank investment projects within recipient countries is mined from AidData’s World Bank Geocoded Research Release (see S1 Table). Investment projects are the dominant type of assistance across development institutions, including the World Bank. The other two types are budget support loans (or “development policy lending” in the current parlance of the Bank), which constitute, on average, about 25 percent of the World Bank’s yearly lending portfolio; and Program-for-Results financing, an instrument which was only developed recently. The analysis in this paper is focused on the traditional and still dominant form of development assistance, investment projects. The database lists 1,183 World Bank investment projects approved in the sample of 58 countries between 2005 and 2014. Taken together, these projects account for commitments of constant 2011 US$ 111.8 billion. The dataset does not provide a geographic breakdown of the overall amount of project commitments; however, the entries in the database typically contain information on the locations (administrative areas) where (part of) a project takes place. We use this information to assign the project locations to the first-level administrative areas (1,084 in our sample of 58 countries) and split total project commitments equally across the subnational administrative areas in which each project is active. We also apply a population-weighted split across the administrative areas as a robustness test.

Subsequently, the share of World Bank funding—measured as commitments of investment projects—each area receives and the share of the nationwide bottom 40 population located in each subnational area are calculated. Simple bivariate correlations between the two variables are calculated to assess the geographical allocation of World Bank project funding within a country. While the World Bank projects variable runs from 2005–14, survey data on the distribution of the bottom 40 are more scarce and only available for some years. In order to mitigate endogeneity concerns, we use, whenever possible, surveys at the beginning of the period under consideration (from 2004 onwards), prioritizing older surveys. We list the survey year for each country in S2 Table (most surveys—13—are from 2007).

These initial calculations of bivariate correlations do not take into account other factors mentioned in section 2 that may influence the targeting of aid. Nonetheless, high positive correlations are taken as an indication of proper geographic targeting insofar as a high proportion of resources go to those areas with a high number of the bottom 40.

To account for other factors affecting the allocation of World Bank funding within countries, in particular the population size of the areas examined, regressions are estimated using, as the dependent variable, the share of World Bank funding each area receives. Zero-inflated beta regressions are undertaken because beta distributions are well suited in the case of continuous variables bound between zero and one. In particular, beta distributions are characterized by high flexibility, thereby allowing varying degrees of skewness. Furthermore, the model is able to account for a not insignificant share of zero values (i.e., areas that do not receive any investment project funding during the period of observation). More precisely, the zero-inflated beta regression distinguishes two separate processes: the first estimates the probability of a value of zero. The underlying idea here is that there is something qualitatively different about administrative areas which receive World Bank funding compared to those which do not. The second process determines, for the areas that receive funding, how much funding these areas receive (see e.g. [29] for more details). A fractional logit model might have also been estimated [30]. However, unlike a beta regression, it would not generate an estimate of a separate process for the probability of the value of zero. Nonetheless, fractional logit models have been estimated to ensure robustness (see S5 Table).

The estimation equation is:

$$y_{ic}=\beta *{ln\;B40}_{ic}+\theta ´X+u_{c}+\varepsilon_{ic},$$ (1)

where *y_ic_* is the share of World Bank funding going to area *i* in country *c*; *ln B*40_*ic*_ is the logarithm of the share of the country’s bottom 40 living in area *i* of country *c*; *X* is a set of control variables; *u_c_* represents country fixed effects; and *ε_ic_* is an idiosyncratic error term. Note that using the log of the share of the national bottom 40 or the log of the number of the bottom 40 living in an area is econometrically equivalent once one controls for country fixed effects. Standard errors are clustered at the country level.

A control is applied for population to identify the effect of the share of the bottom 40 living in an administrative area independent of the population size of the area. In other words, it allows us to determine whether poorer administrative areas receive more or less project funding from the World Bank. Travel time from the administrative areas to the capital is used to control for the ease of access to the administrative areas. The travel time by road (with private transportation) is constructed from estimates gathered through Internet searches. As discussed in section 2, cost-effectiveness considerations may lead to an allocation of resources that neglects—justifiably as donors may argue—the bottom 40 in remote, difficult-to-access areas. In this sense, we may expect the coefficient of the bottom 40 to reflect a more pro-poor allocation once we control for the ease of access of areas. In addition, we include a dummy variable equal to one (zero) if the capital is (not) located in the area. This variable may reflect political economy considerations in terms of the visibility of projects, or, equally possible, efficiency or general equilibrium considerations. Conflict-related deaths per 100,000 inhabitants are used to gauge security and the risks in administrative areas more generally. Given that conflict-prone areas are typically poorer than safer places, we may see a more pro-poor allocation of project funding once we control for risk considerations. Finally, government expenditures and the aid of other donors—only available in a limited sample of 15 and 11 countries, respectively—are included in robustness tests because the World Bank may take government budgetary allocations in administrative areas and the area-based activities of other donors into account in determining its own subnational resource allocations. If an (poor) administrative area is relatively overfunded by the government or other donors, it may be a reasonable decision to take by the World Bank to concentrate on other (less poor) areas. Again, the inclusion of these variables may reveal a more pro-poor allocation of World Bank funding. S1 Table provides definitions and data sources of all the variables used in the empirical analysis.

## Results

### Correlation results

In the correlation analysis, we assess the geographical allocations of World Bank investment projects at one administrative level below the national level against the distribution of the national bottom 40 population across the same subnational administrative levels. In this analysis, an equal split of total project commitments is assumed across the subnational areas in which projects are active. The correlations in most countries tend to be somewhat higher if a population-weighted split of resources across subnational areas is applied (see S2 Table). The results in Table 1 show that, in 41 countries (71 percent), the allocation of World Bank funds is positively correlated with the distribution of the bottom 40 population. In 19 of these countries (33 percent of the total), the correlation is at least 0.5, while the average over all countries is 0.25.

**Table 1:** Subnational allocations, correlation coefficients between the share of World Bank project funding and the bottom 40, by country.

**Correlation**	**Countries**
0.5 to 1.0	Armenia, Bangladesh, Bolivia, Bosnia and Herzegovina, Brazil, Cabo Verde, Chile, El Salvador, Ethiopia, India, Indonesia, Kyrgyz Republic, Lesotho, Madagascar, Mauritania, Nepal, Tajikistan, Uruguay, Republic of Yemen
0 to 0.5	Afghanistan, Angola, Bhutan, Burkina Faso, Cameroon, Chad, Ecuador, Guatemala, Haiti, Iraq, Kenya, Lao People’s Democratic Republic, Mali, Niger, Peru, Philippines, Republic of Congo, Russian Federation, Sri Lanka, Uganda, Ukraine, Vietnam
−0.5 to 0	Belarus, Burundi, Democratic Republic of Congo, Georgia, Ghana, Guinea-Bissau, Mexico, Mozambique, Nigeria, Senegal, South Africa, Tanzania, Timor-Leste
−1.0 to −0.5	Guinea, Rwanda, Sierra Leone, Zambia

*Source*: Estimates based on Global Monitoring Database (internal database), Poverty and Equity Global Practice, World Bank, Washington, DC; World Bank Geocoded Research Release (database), AidData, College of William and Mary, Williamsburg, VA, http://aiddata.org/data/world-bank-geocoded-research-release-level-1-v1-4-2.

However, Table 1 also shows that World Bank allocations in 17 countries are negatively correlated with the bottom 40. This finding raises questions about the allocations of investment projects in these countries that do not seem to target or reach the bottom 40. Some of these results are explained by substantial allocations of project resources to capital cities, whereas most of the bottom 40 live elsewhere. Closer inspection suggests that this is the case mostly in African countries, particularly the Democratic Republic of Congo, Ghana, Guinea, Guinea-Bissau, Mozambique, Senegal, Sierra Leone, Tanzania, and Zambia. In the remaining countries, a negative correlation seems not to stem from the overabsorption of resources by capital cities, but from the higher share of resources going, on average, to areas with lower shares of national bottom 40 populations.

**Fig 1:**
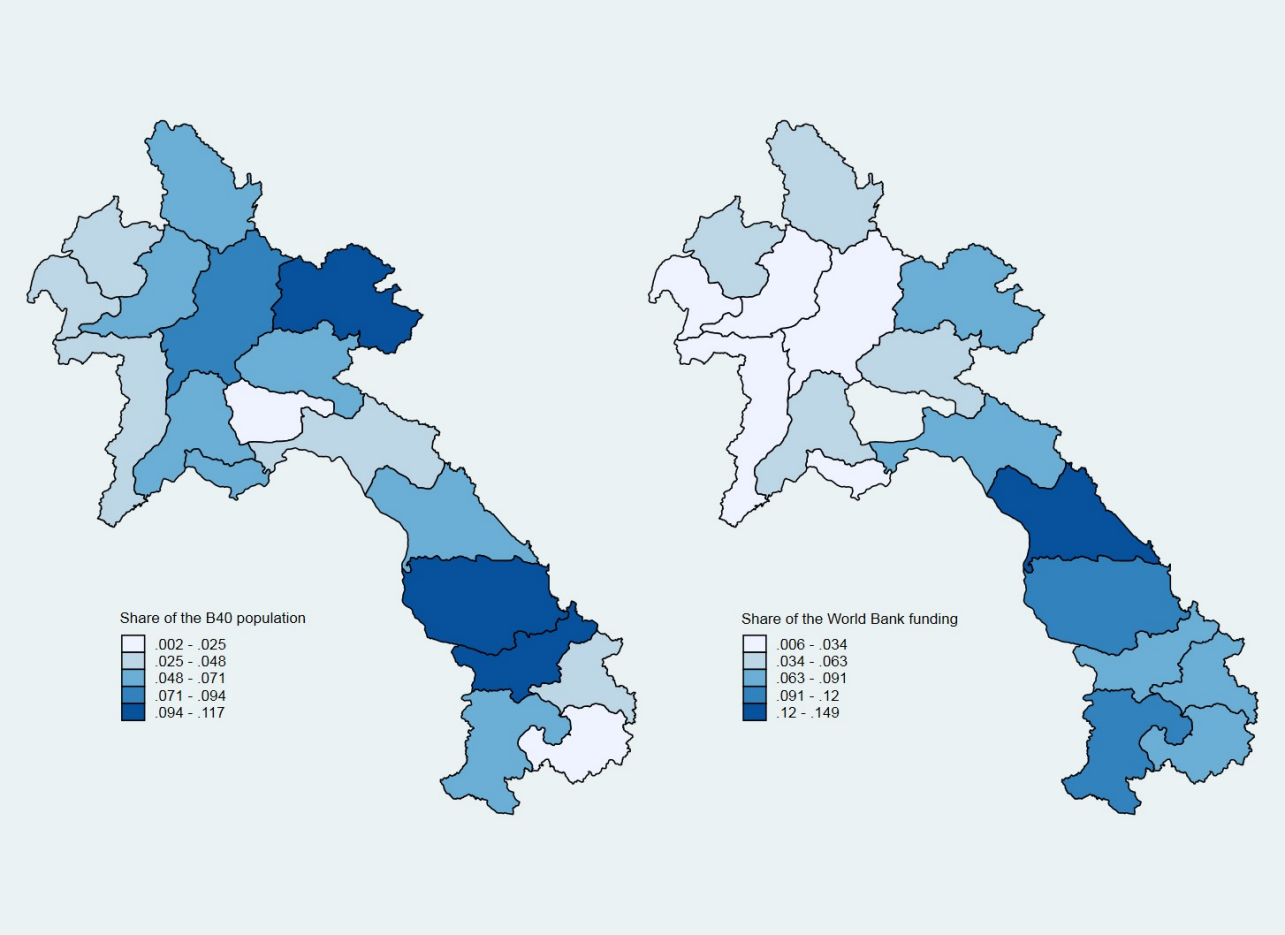
The Distribution of the bottom 40 and World Bank project funding, Lao PDR. *Source*: Estimates based on Global Monitoring Database (internal database), Poverty and Equity Global Practice, World Bank, Washington, DC; World Bank Geocoded Research Release (database), AidData, College of William and Mary, Williamsburg, VA, http://aiddata.org/data/world-bank-geocoded-research-release-level-1-v1-4-2.

To illustrate, Fig 1 displays the geographical distribution of the bottom 40 and World Bank project funding across administrative areas in the Lao People’s Democratic Republic. This allows a simple visualization of the disproportion in the shares of World Bank funding relative to the shares of the bottom 40 in these areas. Although the correlation between the locations of the bottom 40 and of World Bank funding is positive (0.34; see S2 Table), the map clearly highlights the imperfect targeting on the bottom 40. Areas in the north are especially underfunded relative to their shares of the bottom 40.

In two countries for which data are available, robustness tests at the second administrative level (districts) confirm the positive correlations. In Bangladesh, the correlation (0.81) is higher at the district level than at the first administrative level (0.50); whereas in Nepal, the correlation appears considerably weaker (0.39) at the district level than at the first administrative level (0.93).

In Table 2, we assess, for each world region, the geographic correlation between the location of the bottom 40 and investment projects. The average correlation coefficient is lowest in Sub-Saharan Africa (0.04) where almost half of the countries in the sample are located (27). This is much lower than the 0.25 average across the entire sample. At the other extreme, the geographic correlation is highest in the 10 countries covered in Latin America and the Caribbean (0.56). The correlations in the other regions fall between these values.

**Table 2:** Subnational allocations, correlation coefficients between the share of World Bank funding and the bottom 40, by world region.

Region	Countries, number	Country areas, number	Average areas per country	Correlation coefficients		
				Simple average	Weighted average by country population	Weighted average by country commitments
SSA	27	437	16	0.04	0.05	0.10
EAP	5	144	29	0.20	0.42	0.29
ECA	8	148	19	0.38	0.12	0.20
LAC	10	193	19	0.56	0.49	0.66
MENA	2	39	20	0.42	0.38	0.42
SAR	6	123	21	0.45	0.69	0.65
World	58	1,084	19	0.25	0.43	0.39

*Note*: EAP = East Asia and Pacific. ECA = Eastern Europe and Central Asia. LAC = Latin American and the Caribbean. MENA = Middle East and North Africa. SAR = South Asia. SSA = Sub-Saharan Africa.

*Source*: Estimates based on Global Monitoring Database (internal database), Poverty and Equity Global Practice, World Bank, Washington, DC; World Bank Geocoded Research Release (database), AidData, College of William and Mary, Williamsburg, VA, http://aiddata.org/data/world-bank-geocoded-research-release-level-1-v1-4-2.

The regional ranking changes substantially if the countries are weighted by national population or the amount of project funding allocated to each country. The population-weighted average in Latin America and the Caribbean is dragged downward by populous Mexico, which presents a slightly negative correlation coefficient (−0.04). South Asia’s targeting proves far better when weighted by either population or commitment amounts thanks to the high correlation shown in India (0.73). Russia (0.03) is mostly responsible for a large drop in the weighted average coefficients for Eastern Europe and Central Asia.

**Fig 2:**
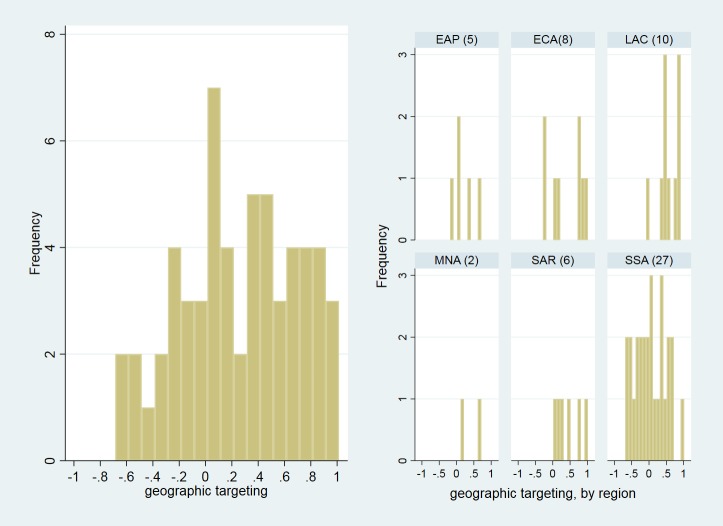
Histograms of correlation coefficients. **a. for the entire sample and b. for each world region.** *Note*: EAP = East Asia and Pacific. ECA = Eastern Europe and Central Asia. LAC = Latin American and the Caribbean. MENA = Middle East and North Africa. SAR = South Asia. SSA = Sub-Saharan Africa. *Source*: Estimates based on Global Monitoring Database (internal database), Poverty and Equity Global Practice, World Bank, Washington, DC; World Bank Geocoded Research Release, (database), AidData, College of William and Mary, Williamsburg, VA, http://aiddata.org/data/world-bank-geocoded-research-release-level-1-v1-4-2.

Fig 2 displays a more disaggregated view of the distribution of the values of the correlation coefficients both for the entire sample (panel a) and for each region (panel b). This reveals mostly positive values in the correlation coefficients across the sample and a broadly uniform distribution in the case of Sub-Saharan Africa, ranging from −0.6 to 0.7, with an outlier at 0.9 (Ethiopia).

In Table 3, we present correlation coefficients for each country income group (according to the 2004 World Bank classification). In addition to the bottom 40, we also calculate correlations with the bottom 20 and bottom 10, which may be more relevant for middle-income countries (see section 2). A first finding is that the correlation coefficients are lowest in low-income countries independently of whether the distribution of bottom 40, 20 or 10 are considered. This is due to the fact that many countries in Sub-Saharan Africa with a low or even negative correlation fall into this category. A second notable finding is that the smaller the part of the income distribution that is taken into account the lower the correlation coefficients are. In fact, in the overall sample the average correlation diminishes from 0.25 (the bottom 40) to 0.20 (the bottom 20) to 0.16 (the bottom 10). As discussed in section 2, we may see a higher correlation of World Bank funding with the bottom 20 or bottom 10 compared to the correlation with the bottom 40 in middle income countries if donors are primarily aiming at eradicating extreme poverty. Although this is not what we find, the results show that the decreasing correlation from the bottom 40 to the bottom 10 is somewhat less pronounced in middle-income countries.

**Table 3:** Subnational allocations, correlation coefficients between the share of World Bank funding and the bottom 40, 20, and 10, by income group.

Income group	Countries, number	Correlation with the distribution of the		
		bottom 40	bottom 20	bottom 10
Low-income countries	35	0.17	0.11	0.08
Lower-middle-income countries	17	0.40	0.37	0.32
Upper-middle-income countries	5	0.32	0.29	0.28
Total	58	0.25	0.20	0.16

*Source*: Estimates based on Global Monitoring Database (internal database), Poverty and Equity Global Practice, World Bank, Washington, DC; World Bank Geocoded Research Release (database), AidData, College of William and Mary, Williamsburg, VA, http://aiddata.org/data/world-bank-geocoded-research-release-level-1-v1-4-2.

Finally, we take into account that geographic targeting of aid is arguably most critical in countries where the geographic income distribution is highly uneven as discussed in section 2. We calculate, for each country, a GINI coefficient of the distribution of the bottom 40 and form quintiles of countries accordingly. The results in S3 Table show that, on average, the more unevenly the bottom 40 are distributed in a country the higher the correlation between World Bank project funding and the bottom 40 is (except for the second quintile of countries, where we find, on average, a negative correlation). Note, however, that this is most probably due to how we construct our correlation coefficients since the share of the national bottom 40 living in an administrative area is typically positively correlated with the total population in that area.

### Regression results

Table 4 presents the results of zero-inflated beta regressions in which the share of World Bank funding an administrative area receives is the dependent variable. Country fixed effects are included to assess within-country correlations between the share of bottom 40 and World Bank funding. The estimates in column 1 do not include any control variables and are therefore analogous to the correlation coefficients described above. Indeed, the positive and significant coefficient of the bottom 40 is in line with the positive correlations between the bottom 40 and World Bank funding found in most countries. The results show that World Bank funding is predominantly allocated to the subnational administrative areas in which most of the bottom 40 reside. Quantitatively, an increase in the average share of the bottom 40 by one percentage point leads, on average, to an increase in the share of World Bank funding by 0.2 percentage points. The effect is rather small because it constitutes only 3.7 percent of the mean of the dependent variable (0.054).

**Table 4:** Zero-inflated beta regressions.

	(1)	(2)	(3)	(4)
Ln bottom 40	0.238***	-0.236***	-0.149**	-0.150**
	(0.035)	(0.060)	(0.062)	(0.060)
Ln population		0.679***	0.547***	0.555***
		(0.080)	(0.090)	(0.088)
Capital			0.290*	0.332**
			(0.175)	(0.136)
Ln travel time			-0.010	
			(0.022)	
Conflict-related deaths			-0.000	
			(0.000)	
Number of countries	58	58	58	58
Number of observations (regions)	1,081	1,081	1,056	1,081

*Note*: The dependent variable is the share of World Bank funding an administrative area receives. Country fixed effects are included in all estimations. Standard errors clustered at the country level are shown in parentheses.

***p < .01

**p < .05

*p < 0.1

In column 2, population is included as a control variable. As expected, the coefficient on population turns out to be significant and positive. The coefficient on the bottom 40 switches sign and becomes significantly negative. The sign switch is visually presented in S1 Fig, which shows the partial residual plots with respect to the bottom 40 for linear regressions with and without controlling for population, which are analogous to the zero-inflated beta estimations in columns 1 and 2 of Table 4.

Note that the correlation between the bottom 40 and population conditional on the country fixed effects is 0.83. The results clearly show that more World Bank funding is allocated to areas with larger populations, which thus also tend to comprise larger shares of the bottom 40. If one controls for population, a higher share of the bottom 40 becomes associated with less World Bank funding. Quantitatively, an increase in the average share of the bottom 40 by one percentage point leads, on average, to a decrease in the share of World Bank funding by 0.2 percentage points (3.6 percent of the mean). This finding shows that the subnational allocation of World Bank project funding is not oriented toward poorer areas within countries in terms of the share of the bottom 40 in the population of the areas because these areas receive, on average, less funding from the World Bank.

The estimates in column 3 add three more control variables: a dummy variable equal to one if the capital city is located in the respective area, the estimated travel time from the other administrative areas to the capital, and the number of conflict-related deaths. The only significant one among these is the dummy variable for the capital city. The finding shows that areas with a capital city tend to receive a higher share of World Bank funding independently of the size of the population or the share of the bottom 40. Since capital areas are typically richer than other areas, i.e., the share of the bottom 40 in their populations appear to be relatively small, the negative coefficient of the share of the bottom 40 becomes smaller when we control for the capital area. It appears that the adverse relationship between poorer areas and World Bank aid is to some extent explained by the fact that the World Bank tends to concentrate its projects in the capitals. Efficiency, general equilibrium, or visibility considerations may play a role (see section 2). At the same time, the observed poverty orientation of World Bank allocations is not altered when we take cost-effectiveness and risk considerations into account by controlling for travel time to the capital and conflict-related deaths.

In column 4, the insignificant variables—travel time and conflict-related deaths—are excluded. Apart from efficiency gains because of fewer explanatory variables, the number of observations (areas) rises relative to column (3) because the data on estimated travel times to the capital are not available for all areas. The significance level of the capital area increases from ten percent in column (3) to five percent in column (4). In quantitative terms, the fact that an area encompasses the capital city raises the share of World Bank funding the area receives, on average, by 1.6 percentage points (29.7 percent of the mean). This substantial effect indicates that donors are inclined to work in capital cities.

Table 5 shows the results across world regions. Standard errors are not clustered in these regressions because of the relatively small number of clusters (countries). Robust standard errors are estimated, however. In columns 1–5, only the bottom 40 variable is included, whereas the estimates in columns 6–10 add population and the capital area as control variables. The estimates with only the bottom 40 variable show that the coefficient on the bottom 40 is, although positive and significant at the five percent level, substantially smaller in the case of Sub-Saharan Africa than in other regions. The estimates on the Middle East and North Africa are not included because only two countries (with 39 subnational areas) in that region are included in the sample. The inclusion of the two control variables changes the picture substantially. While the coefficient on the bottom 40 is significant at the one percent level and negative in Sub-Saharan Africa, it is not significant in the other regions except Latin America and the Caribbean. In this region, the coefficient turns out significant at the five percent level and positive. Hence, the negative and significant coefficient in columns 3 and 4 of Table 3 appear to be solely driven by the subnational allocation of World Bank project funding in Sub-Saharan Africa. There, poorer subnational areas appear to receive less project funding, whereas this is seemingly not the case in the other world regions. In Latin America and the Caribbean, it even appears that poorer areas receive *more* World Bank project funding. An additional finding is that the coefficient on the capital area is only significant in South Asia and Sub-Saharan Africa.

**Table 5:** Zero-inflated beta regressions, by world region.

	(1)	(2)	(3)	(4)	(5)	(6)	(7)	(8)	(9)	(10)
	SSA	EAP	ECA	LAC	SAR	SSA	EAP	ECA	LAC	SAR
Ln bottom 40	0.095**	0.270***	0.511***	0.382***	0.318***	-0.214***	0.017	-0.102	0.306**	-0.119
	(0.040)	(0.082)	(0.114)	(0.063)	(0.043)	(0.058)	(0.144)	(0.255)	(0.152)	(0.115)
Ln population						0.626***	0.365**	0.688**	0.041	0.653***
						(0.078)	(0.172)	(0.279)	(0.197)	(0.169)
Capital						0.593***	0.450	-0.292	0.408	0.378**
						(0.144)	(0.461)	(0.279)	(0.365)	(0.189)
Number of countries	27	5	8	10	6	27	5	8	10	6
Number of observations	436	144	148	193	121	436	144	148	193	121

*Note*: The dependent variable is the share of World Bank funding a region receives. Country fixed effects are included in all estimations. Robust standard errors are shown in parentheses. EAP = East Asia and Pacific. ECA = Eastern Europe and Central Asia. LAC = Latin America and the Caribbean. SAR = South Asia. SSA = Sub-Saharan Africa.

***p < .01

**p < .05

*p < 0.1.

Table 6 reports results for two categories of sectors: According to the World Bank (2009), in particular education and health care services should be provided to people in need independently of their location in a country because of the goal of universal coverage in these sectors. In transportation, energy, and mining projects, on the other hand, subnational allocation decisions depend on practical considerations such as the availability of natural waterways or raw materials. The results of the regressions without population in columns 1 and 4 indicate that the coefficients for the two categories of projects are virtually the same size. This means that funding for education and health care projects is not disproportionally allocated to areas with higher numbers of the bottom 40 relative to the funding for transportation, energy, and mining projects.

**Table 6:** Zero-inflated beta regressions, education and Health versus transportation, energy, and mining.

	(1)	(2)	(3)	(4)	(5)	(6)
	Education and health projects			Transportation, energy and mining projects		
Ln bottom 40	0.144***	0.037	0.006	0.149***	-0.129**	-0.143**
	(0.030)	(0.063)	(0.063)	(0.040)	(0.064)	(0.068)
Ln population		0.155**	0.202**		0.431***	0.431***
		(0.078)	(0.078)		(0.086)	(0.089)
Capital		-0.149	0.068		0.301*	0.276
		(0.156)	(0.182)		(0.169)	(0.199)
Ln travel time			0.050**			-0.007
			(0.023)			(0.028)
Conflict-related deaths			-0.0003			-0.0005**
			(0.0003)			(0.0002)
Number of countries	51	51	51	54	54	54
Number of observations (areas)	964	964	946	999	999	974

*Note*: The dependent variable is the share of World Bank funding a region receives. Only countries with projects in the respective sectors are included. Country fixed effects are included in all estimations. Standard errors clustered at the country level are shown in parentheses.

***p < .01

**p < .05

*p < 0.1.

However, as columns 2 and 5 show, a different picture emerges after controls are applied for population and the capital area. While the coefficient on the bottom 40 is not significant in the case of education and health care projects, it becomes negative and significant at the five percent level for transportation, energy, and mining projects. The negative coefficients of such projects may be explained by general equilibrium effects because projects located in areas with a smaller share of the poor may still exert high general equilibrium impacts on the poor. The estimates in column 3 show some evidence that education and health care projects tend to be located in more remote areas. According to column 6, transportation, energy, and mining projects appear, meanwhile, to be located less frequently in conflict-affected areas.

Table 7 includes public expenditure by recipient governments and the aid of other donors as additional control variables. Recall that the inclusion of these variables may reveal a more pro-poor allocation of World Bank funding because the Word Bank may take into account that some (poor) administrative areas are sufficiently supported by the government or other donors. The number of countries is reduced to 15 (public expenditure) and 11 (aid from other donors) because of data availability constraints. This affects the significance levels of the bottom 40 variable, and the variable even becomes insignificant in some cases. Rather surprisingly, the coefficient switches sign and becomes negative and significant at the ten percent level (compared to column 1 of Table 4) when we account for the allocations of other donors in column 5. At the same time, aid of other donors is positively and significantly correlated with World Bank funding. Hence, there is no evidence of donor coordination. On the contrary, the analysis shows evidence of geographic clustering by the World Bank and the other donors. The conformity in location choices may yield important benefits for donor organizations linked to logistics, security, and leverage (see section 2). Note that, when we re-run these regressions without controlling for aid of other donors, but with the same sample of countries (not shown), the results reveal that the changes in the coefficients of the bottom 40 are due to the reduced sample and not the inclusion of aid of other donors as an additional explanatory variable. Table 7 also shows that public expenditure by national governments seems not to affect World Bank allocations.

**Table 7:** Zero-inflated beta regressions, public expenditure and the aid of other donors.

	(1)	(2)	(3)	(4)	(5)	(6)	(7)	(8)
Ln bottom 40	0.128*	-0.075	-0.016	0.041	-0.132*	-0.398***	-0.246***	-0.254***
	(0.071)	(0.085)	(0.081)	(0.076)	(0.074)	(0.093)	(0.093)	(0.090)
Ln population		0.402***	0.335***	0.236***		0.775***	0.491***	0.483***
		(0.111)	(0.099)	(0.075)		(0.131)	(0.150)	(0.161)
Capital			0.269*	-0.128			0.800***	1.109***
			(0.140)	(0.208)			(0.248)	(0.420)
Ln travel time				-0.104*				0.059
				(0.055)				(0.061)
Conflict-related deaths				0.0001				-0.0011
				(0.0001)				(0.0007)
Log public expenditure	0.156	-0.014	-0.038	-0.061				
	(0.113)	(0.112)	(0.104)	(0.097)				
Log aid of other donors					0.231***	0.144***	0.142***	0.150***
					(0.061)	(0.041)	(0.041)	(0.053)
Number of countries	15	15	15	15	11	11	11	11
Number of observations (areas)	320	320	320	313	267	267	267	262

*Note*: The dependent variable is the share of World Bank funding a region receives. Country fixed effects are included in all estimations. Standard errors clustered at the country level are shown in parentheses.

***p < .01

**p < .05

*p < 0.1.

In a robustness test, we use the share of the bottom 20 or bottom 10 instead of the bottom 40 as our variable of interest (S4 Table). We find smaller positive coefficients of the bottom 20 and bottom 10 compared to that of the bottom 40 when we do not control for any other variables in the regressions (columns 1–3). At the same time however, the results show smaller negative coefficients of the bottom 20 and bottom 10 compared to that of the bottom 40 when we apply population and capital area as controls (columns 4–6). Thus, it seems that World Bank allocations are less affected by the bottom 20 and bottom 10—in absolute as well as in relative terms—compared to the bottom 40.

In a series of other robustness tests, certain groups of countries are excluded; the observations are weighted in various ways; the dependent variable is altered; and the estimation method is changed (S5 Table). First, the quintile of countries with the most even geographical distribution of the bottom 40 (according to the Gini coefficient) is excluded from the estimation since one may argue that effective geographical targeting is relatively unimportant in countries with a relatively uniform income distribution across administrative areas. Second, countries with five or fewer first-level administrative areas are excluded from the regression (five countries). Third, countries in which only five or fewer World Bank projects have been conducted over 1995–2004 are excluded (six countries). Fourth, the observations are weighted so that each country, rather than each administrative area, has the same weight in the regression. Fifth, the construction of the dependent variable is altered, and the subnational administrative areas are weighted by population (rather than equally) in splitting total project commitments across the areas in which a project is active. Sixth, the amount of World Bank funding is used as the dependent variable instead of the share of funding an area receives, and a Poisson regression model is estimated. Finally, a fractional logit model is estimated instead of a zero-inflated beta model. Recall that both are valid estimation methods in the case of a continuous variable bound between zero and one as the dependent variables. The results remain qualitatively the same as the results shown in Table 3 except that the coefficient on the dummy variable for the capital area becomes insignificant in some cases.

## Conclusion

This paper focuses on the second corporate goal of the World Bank, i.e., shared prosperity, and assesses the geographic targeting by correlating the geographical allocation of World Bank funding within countries with the geographical distribution of the bottom 40 as measured by survey income or consumption data. The correlation analysis indicates that, of the 58 countries in the sample, 41 show a positive correlation between the shares of the bottom 40 and World Bank funding, and almost half of these countries show a correlation above 0.5. Slightly more than a quarter of the countries in the total sample, mostly in Sub-Saharan Africa, exhibit a negative correlation. Indeed, the geographic correlation between the bottom 40 and World Bank investment funding is lowest in Sub-Saharan Africa, with an average correlation coefficient of almost zero, which is significantly lower relative to the entire sample. This result can be explained by the substantial allocation of project resources to the capitals in many Sub-Saharan Africa countries, whereas most of the bottom 40 live elsewhere.

The presence of the bottom 40 is, however, typically correlated with the size of the population in an administrative area. The regression analysis shows that, once one controls for population, the correlation between the bottom 40 and World Bank funding switches sign and becomes significant and negative on average. This is entirely driven by Sub-Saharan Africa and not observed in the other regions. Hence, the significant and positive correlation in the estimates without controls for population indicates that World Bank project funding is concentrated in areas in which more people live, including the bottom 40, rather than in poorer areas.

Several factors may explain the imperfect targeting of aid to the bottom 40. In the regression analysis, we control for factors such as remoteness, conflicts, and domestic and other external financing, which may constitute valid reasons for donors not to allocate aid to where the bottom 40 live. First, cost-effectiveness considerations may lead to an allocation of resources that neglects the poor in remote, difficult-to-access areas. Similarly, risk considerations may keep donors from engaging in conflict-affected poorer areas. Furthermore, the World Bank may take government budgetary allocations in administrative areas and the area-based activities of other donors into account in determining its own subnational resource allocations. If an (poor) administrative area is relatively overfunded by the government or other donors, it may be a reasonable decision to take by the World Bank to concentrate on other (less poor) areas. The inclusion of these variables may reveal a more pro-poor allocation of World Bank funding.

The regression analysis reveals that the only variable that alters the observed relationship between project locations and the locations of the bottom 40 is the dummy variable for capital cities. It appears that the negative relationship between poorer areas and World Bank funding is to some extent explained by the fact that the World Bank tends to concentrate its projects in the capitals. The interpretation of this finding is not clear. Political economy considerations in terms of the visibility of projects on the one hand, and efficiency or general equilibrium considerations on the other hand may constitute the explanations for this finding. Country case studies would be required to identify the context-specific reasons for the allocation patterns in individual countries. Nevertheless, the finding that the allocation of education and health care projects is more oriented towards poorer areas than that of energy, mining, and transportation projects is consistent with the proposition that the World Bank takes into account general equilibrium effects.

Given that the interpretation of low or negative (partial) correlations may be ambiguous, such findings may be seen as a burden of proof on donors to explain their allocation decisions rather than as negative assessments. With respect to its second corporate goal, the World Bank ought to monitor more closely the extent to which the geographical distribution of project funding and the location of the bottom 40 align. Such a monitoring exercise has the potential to enhance substantially the efficiency of increasingly scarce development funds and help maximize the poverty-reducing effects of development assistance. Similar studies of other development banks and large bilateral donor agencies aimed not only at investigating spatial poverty targeting, but also at other development goals would also be welcome.

## Supporting information

S1 Fig Partial residual plots, bottom 40, linear regressions. (DOCX)

S1 Table Definitions of variables and data sources. (DOCX)

S2 Table Subnational allocations, correlation coefficients, by country. (DOCX)

S3 Table Subnational allocations, correlation coefficients between the share of World Bank funding and the bottom 40, by GINI quintile. (DOCX)

S4 Table Zero-inflated beta regressions, bottom 40, 20, or 10. (DOCX)

S5 Table Further robustness tests. (DOCX)

## References

[1] Nations United. 2015 Resolution adopted by the General Assembly on 25 September 2015. Available from: http://www.un.org/ga/search/view_doc.asp?symbol=A/RES/70/1&Lang=E.

[2] Lakner C, Negre M, and Prydz EB. Twinning the Goals: How Can Shared Prosperity Help to Reduce Global Poverty? Policy Research Working Paper. 2014; 7106. World Bank. Available from: http://documents.worldbank.org/curated/en/816921468339602084/Twinning-the-goals-how-can-promoting-shared-prosperity-help-to-reduce-global-poverty.

[3] World Bank. Poverty and Shared Prosperity 2016: Taking on Inequality. Washington, DC: World Bank; 2016.

[4] Elbers C, Fujii T, Lanjouw P, Özler B, Yin W. Poverty Alleviation through Geographic Targeting: How Much Does Disaggregation Help? Journal of Development Economics. 2007;83: 198–213.

[5] Fujii T. How Well Can We Target Aid with Rapidly Collected Data? Empirical Results for Poverty Mapping from Cambodia. World Development. 2008;36: 1830–1842.

[6] Karlan DS., Thuysbaert B. Targeting Ultra-Poor Households in Honduras and Peru. World Bank Economic Review. 2019; 33: 63–94.

[7] De R, Becker C. 2015. The Foreign Aid Effectiveness Debate: Evidence from Malawi. AidData Working Paper. 2015;6. AidData. Available from: https://www.aiddata.org/publications/the-foreign-aid-effectiveness-debate-evidence-from-malawi.

[8] Nunnenkamp P, Öhler H, Sosa Andrés M. Need, Merit, and Politics in Multilateral Aid Allocation: A District‐Level Analysis of World Bank Projects in India. Review of Development Economics. 2017;21: 126–56.

[9] Odokonyero T, Ijjo A, Marty R, Muhumuza T, Moses GO. The impact of aid on health outcomes in Uganda. Health Economics. 2018;27: 733–745. 10.1002/hec.3632 29271088

[10] Briggs RC. Does Foreign Aid Target the Poorest? International Organization. 2017;71: 187–206.

[11] Briggs RC. Poor Targeting: A Gridded Spatial Analysis of the Degree to Which Aid Reaches the Poor in Africa. World Development. 2018;103: 133–148.

[12] Öhler H, Nunnenkamp P. Needs-Based Targeting or Favoritism? The Regional Allocation of Multilateral Aid within Recipient Countries. Kyklos. 2014;67: 420–46.

[13] Engstrom R, Hersh J, Newhouse D. Poverty from Space: Using High-Resolution Satellite Imagery for Estimating Economic Well-Being. World Bank Policy Research Working Paper. 2017; 8284. World Bank. Available from: http://documents.worldbank.org/curated/en/610771513691888412/Poverty-from-space-using-high-resolution-satellite-imagery-for-estimating-economic-well-being.

[14] Jean N, Burke M, Xie M, Davis WM, Lobell D, Ermon S. Combining Satellite Imagery and Machine Learning to Predict Poverty. Science. 2016; 353:790–94. 10.1126/science.aaf7894 27540167

[15] Clements MA., Kremer M. The New Role for the World Bank. Journal of Economic Perspectives. 2016;30: 53–76. 28443650

[16] Carter P. 2014 Aid Allocation Rules. European Economic Review. 71: 132–51.

[17] Christiaensen L, Weerdt JD, Kanbur R. Cities, Towns, and Poverty: Migration Equilibrium and Income Distribution in a Todaro-Type Model with Multiple Destinations. IZA Discussion Paper. 2017;10692. Institute for the Study of Labor. Available from: https://www.iza.org/de/publications/dp/10692/cities-towns-and-poverty-migration-equilibrium-and-income-distribution-in-a-todaro-type-model-with-multiple-destinations.

[18] World Bank. World Development Report 2009: Reshaping Economic Geography. Washington, DC: World Bank; 2009.

[19] Öhler H. Do Aid Donors Coordinate Within Recipient Countries? AWI Discussion Paper. 2013;539. Heidelberg University. Available from: http://archiv.ub.uni-heidelberg.de/volltextserver/14372/.

[20] Kirk JA. Banking on India’s States: The Politics of World Bank Reform Programs in Andhra Pradesh and Karnataka. India Review. 2005;4: 287–325.

[21] Dreher A, Fuchs A, Hodler R, Parks BC, Raschky PA, Tierney MJ. Aid on Demand: African Leaders and the Geography of China’s Foreign Assistance. AidData Working Paper. 2016;3. AidData. Available from: https://www.aiddata.org/publications/aid-on-demand-african-leaders-and-the-geography-of-chinas-foreign-assistance.

[22] Feyzioglu T, Swaroop V, Zhu M. A Panel Data Analysis of the Fungibility of Foreign Aid. World Bank Economic Review. 1998;12: 29–58.

[23] Pack H, Pack JR. Foreign Aid and the Question of Fungibility. Review of Economics and Statistics. 1993;75: 258–65.

[24] Van de Sijpe N. Is Foreign Aid Fungible? Evidence from the Education and Health Sectors. World Bank Economic Review. 2013;27: 320–56.

[25] World Bank. World Bank Group: A New Approach to Country Engagement. World Bank; 2014 Available from: http://documents.worldbank.org/curated/en/940631468324888939/pdf/878460BR0R2014050Box385206B00OUO090.pdf.

[26] Deaton A, Zaidi S. Guidelines for Constructing Consumption Aggregates for Welfare Analysis. LSMS Working Paper. 2002;135, World Bank. Available from: https://openknowledge.worldbank.org/handle/10986/14101.

[27] Ferreira FHG, Chen S, Dabalen A, Dikhanov Y, Hamadeh N, Jolliffe D, et al A Global Count of the Extreme Poor in 2012: Data Issues, Methodology and Initial Results. Journal of Economic Inequality. 2016;14: 141–172.

[28] World Bank. PovcalNet: An Online Analysis Tool for Global Poverty Monitoring [cited 5 March 2019]. Available from: http://iresearch.worldbank.org/PovcalNet.

[29] Ospina R, Ferrari SLP. A General Class of Zero-or-One Inflated Beta Regression Models. Computational Statistics and Data Analysis. 2012;56: 1609–23.

[30] Papke LE., Wooldridge JM. Econometric Methods for Fractional Response Variables with an Application to 401(k) Plan Participation Rates. Journal of Applied Econometrics. 1996;11: 619–32.

