# Supplementary material for: Putting your money where your mouth is: Geographic targeting of World Bank projects to the bottom 40 percent
Source: PLoS One. 2019 Jun 21;14(6):e0218671. doi: 10.1371/journal.pone.0218671 (PMC6588237; doi:10.1371/journal.pone.0218671)
Supplement: S1 Fig — (DOCX) [file pone.0218671.s001.docx]

**S1 Fig: Partial residual plots, bottom 40, linear regressions**

**a. Without controlling for population**

**b. Controlling for population**

*Source:* Estimates based on Global Monitoring Database (internal database), Poverty and Equity Global Practice, World Bank, Washington, DC; World Bank Geocoded Research Release, (database), AidData, College of William and Mary, Williamsburg, VA, http://aiddata.org/data/world-bank-geocoded-research-release-level-1-v1-4-2.
