# Supplementary material for: Putting your money where your mouth is: Geographic targeting of World Bank projects to the bottom 40 percent
Source: PLoS One. 2019 Jun 21;14(6):e0218671. doi: 10.1371/journal.pone.0218671 (PMC6588237; doi:10.1371/journal.pone.0218671)
Supplement: S1 Table — (DOCX) [file pone.0218671.s002.docx]

**S1 Table: Definitions of variables and data sources**

| Variable | Definition | Source |
| --- | --- | --- |
| World Bank funding | Total commitments of investment projects of the World Bank in U.S. dollars split equally (or population weighted) across the subnational first-level administrative areas in which a project has been active; 2005–14 | *AidData*  World Bank Geocoded Research Release, (database), AidData, College of William and Mary, Williamsburg, VA (accessed November 23, 2016), http://aiddata.org/data/world-bank-geocoded-research-release-level-1-v1-4-2 |
| Share of World Bank funding | Share of World Bank project funding a subnational administrative area receives | *AidData* |
| Share of the bottom 40 | Share of the national bottom 40 population living in an administrative area; data from surveys within the 2005–14 period | *Global Poverty Monitoring*  Source: Global Monitoring Database (internal database), Poverty and Equity Global Practice, World Bank, Washington, DC (accessed December 2016–January 2017) |
| Ln bottom 40 | Number of the national bottom 40 population living in an area (in log) | *Global Poverty Monitoring* |
| Ln population | Number of people living in an area (in log) | *Global Poverty Monitoring* |
| Capital area | Dummy variable equal to one (zero) if the capital of the country is (not) located in the area | *Internet searches* |
| Ln travel time | Estimated road travel time with private transportation from each subnational area to the respective capital in minutes (in log) | *Internet searches* |
| Conflict-related deaths | Number of conflict-related deaths in an area (per 100,000 inhabitants); 2005–14 | *UCDP*  UCDP (Uppsala Conflict Data Program) (database), Department of Peace and Conflict Research, Uppsala University, Uppsala, Sweden (accessed June 20, 2017), http://ucdp.uu.se/?id=1) http://ucdp.uu.se/downloads/ |
| Ln public expenditure | Subnational public expenditure (in log); 2005–14 or subperiods | *Boost*  Open Budgets Portal: Boost (database), World Bank, Washington, DC (accessed June 20, 2017), http://wbi.worldbank.org/boost/country |
| Ln aid of other donors | Aid of other donors (in log); 2005-2014 (expect in the case of Senegal for which the period covered is 2005-2012) | AidData http://aiddata.org/subnational-geospatial-research-datasets (accessed: September 28^th^, 2017) |
