# Supplementary material for: Putting your money where your mouth is: Geographic targeting of World Bank projects to the bottom 40 percent
Source: PLoS One. 2019 Jun 21;14(6):e0218671. doi: 10.1371/journal.pone.0218671 (PMC6588237; doi:10.1371/journal.pone.0218671)
Supplement: S2 Table — (DOCX) [file pone.0218671.s003.docx]

**S2 Table: Subnational allocations, correlation coefficients, by country**

| Country | Survey year | Administrative areas, number | Correlation coefficients | |
| --- | --- | --- | --- | --- |
|  |  |  | Project locations equally weighted | Project locations weighted by population |
| Afghanistan | 2007 | 34 | 0.20 | 0.12 |
| Angola | 2008 | 18 | 0.20 | 0.37 |
| Armenia | 2009 | 11 | 0.78 | 0.95 |
| Bangladesh | 2005 | 7 | 0.50 | 0.60 |
| Belarus | 2011 | 7 | -0.28 | -0.45 |
| Bhutan | 2007 | 20 | 0.07 | 0.13 |
| Bolivia | 2007 | 9 | 0.85 | 0.95 |
| Bosnia and Herzegovina | 2007 | 3 | 0.96 | 0.98 |
| Brazil | 2012 | 27 | 0.75 | 0.77 |
| Burkina Faso | 2009 | 13 | 0.09 | 0.07 |
| Burundi | 2006 | 17 | -0.26 | -0.21 |
| Cameroon | 2007 | 10 | 0.23 | 0.39 |
| Cabo Verde | 2007 | 22 | 0.62 | 0.55 |
| Chad | 2011 | 20 | 0.02 | 0.05 |
| Chile | 2006 | 13 | 0.86 | 0.95 |
| Congo, Dem. Rep. | 2012 | 11 | -0.16 | 0.03 |
| Congo, Rep. | 2011 | 12 | 0.41 | 0.54 |
| Ecuador | 2007 | 22 | 0.44 | 0.46 |
| El Salvador | 2007 | 14 | 0.57 | 0.91 |
| Ethiopia | 2010 | 11 | 0.92 | 0.97 |
| Georgia | 2008 | 10 | -0.22 | 0.18 |
| Ghana | 2005 | 10 | -0.24 | -0.11 |
| Guatemala | 2006 | 22 | 0.34 | 0.88 |
| Guinea | 2012 | 8 | -0.60 | -0.48 |
| Guinea-Bissau | 2010 | 9 | -0.01 | 0.05 |
| Haiti | 2012 | 10 | 0.46 | 0.78 |
| India | 2009 | 35 | 0.73 | 0.86 |
| Indonesia | 2011 | 33 | 0.64 | 0.92 |
| Iraq | 2006 | 18 | 0.19 | 0.57 |
| Kenya | 2005 | 8 | 0.42 | 0.81 |
| Kyrgyz Republic | 2012 | 8 | 0.85 | 0.97 |
| Lao PDR | 2007 | 18 | 0.34 | 0.62 |
| Lesotho | 2010 | 10 | 0.59 | 0.81 |
| Madagascar | 2010 | 6 | 0.54 | 0.60 |
| Mali | 2009 | 9 | 0.32 | 0.46 |
| Mauritania | 2008 | 13 | 0.69 | 0.73 |
| Mexico | 2008 | 32 | -0.04 | 0.18 |
| Mozambique | 2008 | 11 | -0.35 | 0.17 |
| Nepal | 2010 | 5 | 0.93 | 0.99 |
| Niger | 2011 | 8 | 0.35 | 0.44 |
| Nigeria | 2009 | 37 | -0.05 | 0.17 |
| Peru | 2004 | 25 | 0.43 | 0.63 |
| Philippines | 2006 | 17 | 0.11 | 0.26 |
| Russian Federation | 2007 | 77 | 0.03 | 0.10 |
| Rwanda | 2010 | 5 | -0.57 | -0.13 |
| Senegal | 2011 | 14 | -0.34 | -0.33 |
| Sierra Leone | 2011 | 4 | -0.64 | -0.65 |
| South Africa | 2010 | 9 | -0.17 | 0.17 |
| Sri Lanka | 2009 | 22 | 0.29 | 0.51 |
| Tajikistan | 2009 | 5 | 0.77 | 0.85 |
| Tanzania | 2011 | 21 | -0.39 | -0.17 |
| Timor-Leste | 2007 | 13 | -0.18 | -0.34 |
| Uganda | 2012 | 112 | 0.09 | 0.06 |
| Ukraine | 2007 | 27 | 0.18 | 0.32 |
| Uruguay | 2007 | 19 | 0.90 | 0.97 |
| Vietnam | 2010 | 63 | 0.11 | 0.28 |
| Yemen, Rep. | 2005 | 21 | 0.65 | 0.83 |
| Zambia | 2010 | 9 | -0.54 | -0.43 |

Source: Estimates based on Global Monitoring Database (internal database), Poverty and Equity Global Practice, World Bank, Washington, DC; World Bank Geocoded Research Release (database), AidData, College of William and Mary, Williamsburg, VA, http://aiddata.org/data/world-bank-geocoded-research-release-level-1-v1-4-2.
