# Supplementary material for: Putting your money where your mouth is: Geographic targeting of World Bank projects to the bottom 40 percent
Source: PLoS One. 2019 Jun 21;14(6):e0218671. doi: 10.1371/journal.pone.0218671 (PMC6588237; doi:10.1371/journal.pone.0218671)
Supplement: S3 Table — (DOCX) [file pone.0218671.s004.docx]

S3 Table. Subnational allocations, correlation coefficients between the share of World Bank funding and the bottom 40, by GINI quintile.

| GINI coefficient | Average correlation |
| --- | --- |
| 1st quintile | 0.10 |
| 2nd quintile | -0.13 |
| 3rd quintile | 0.24 |
| 4th quintile | 0.50 |
| 5th quintile | 0.55 |
| Total | 0.25 |

Source: Estimates based on Global Monitoring Database (internal database), Poverty and Equity Global Practice, World Bank, Washington, DC; World Bank Geocoded Research Release (database), AidData, College of William and Mary, Williamsburg, VA, http://aiddata.org/data/world-bank-geocoded-research-release-level-1-v1-4-2.
