# Supplementary material for: Putting your money where your mouth is: Geographic targeting of World Bank projects to the bottom 40 percent
Source: PLoS One. 2019 Jun 21;14(6):e0218671. doi: 10.1371/journal.pone.0218671 (PMC6588237; doi:10.1371/journal.pone.0218671)
Supplement: S4 Table — (DOCX) [file pone.0218671.s005.docx]

S4 Table. Zero-inflated beta regressions, bottom 40, 20, or 10.

|  | (1) | (2) | (3) | (4) | (5) | (6) |
| --- | --- | --- | --- | --- | --- | --- |
| Ln bottom 40 | 0.238*** |  |  | -0.153*** |  |  |
|  | (0.035) |  |  | (0.059) |  |  |
| Ln bottom 20 |  | 0.159*** |  |  | -0.072* |  |
|  |  | (0.033) |  |  | (0.039) |  |
| Ln bottom 10 |  |  | 0.124*** |  |  | -0.054* |
|  |  |  | (0.030) |  |  | (0.032) |
| Ln population |  |  |  | 0.563*** | 0.474*** | 0.458*** |
|  |  |  |  | (0.088) | (0.072) | (0.067) |
| Capital |  |  |  | 0.340** | 0.398*** | 0.362*** |
|  |  |  |  | (0.136) | (0.134) | (0.126) |
|  |  |  |  |  |  |  |
| Observations | 1,081 | 1,072 | 1,047 | 1,080 | 1,071 | 1,046 |

*Note:* The dependent variable is the share of World Bank funding a region receives. Country fixed effects are included in all estimations. Standard errors clustered at the country level are shown in parentheses. ***p < .01, **p < .05, *p < 0.1.
