# Supplementary material for: Putting your money where your mouth is: Geographic targeting of World Bank projects to the bottom 40 percent
Source: PLoS One. 2019 Jun 21;14(6):e0218671. doi: 10.1371/journal.pone.0218671 (PMC6588237; doi:10.1371/journal.pone.0218671)
Supplement: S5 Table — (DOCX) [file pone.0218671.s006.docx]

**S5 Table. Further robustness tests**

|  | (1) | (2) | (3) | (4) | (5) | (6) | (7) |
| --- | --- | --- | --- | --- | --- | --- | --- |
| Ln B40 | -0.165*** | -0.129** | -0.175*** | -0.229*** | -0.191*** | -0.192** | -0.231*** |
|  | (0.063) | (0.061) | (0.055) | (0.069) | (0.063) | (0.075) | (0.060) |
| Ln population | 0.593*** | 0.502*** | 0.609*** | 0.708*** | 1.111*** | 0.748*** | 0.759*** |
|  | (0.094) | (0.083) | (0.083) | (0.126) | (0.101) | (0.116) | (0.086) |
| Capital | 0.253 | 0.395*** | 0.306** | 0.208 | 0.461*** | -0.058 | 0.315** |
|  | (0.164) | (0.136) | (0.129) | (0.159) | (0.152) | (0.132) | (0.140) |
|  |  |  |  |  |  |  |  |
| Number of countries | 46 | 53 | 52 | 58 | 58 | 58 | 58 |
| Number of observations | 1,081 | 1,059 | 979 | 1,081 | 1,081 | 1,081 | 1,081 |

*Note:* The dependent variable is the share of World Bank funding a region receives. In column 1, the quintile of countries with the most even geographical distribution of the bottom 40 (according to the Gini coefficient) is excluded from the estimation. In column 2, countries with five or fewer first-level administrative areas are excluded from the regression (five countries). In column 3, countries in which only five or fewer World Bank projects have been conducted over 1995–2004 are excluded (six countries). In column 4, the observations are weighted so that each country, rather than each administrative area, has the same weight in the regression. In column 5, the construction of the dependent variable is altered, and the subnational administrative areas are weighted by population (rather than equally) in splitting total project commitments across the areas in which a project is active. In column 6, the amount of World Bank funding is used as the dependent variable instead of the share of funding an area receives, and a Poisson regression model is estimated. In column 7, a fractional logit model is estimated instead of a zero-inflated beta model. Country fixed effects are included in all estimations. Standard errors clustered at the country level are shown in parentheses. ***p < .01, **p < .05, *p < .1
